# Predicting the length of volleyball serves: The role of early auditory and visual information

**DOI:** 10.1371/journal.pone.0208174

**Published:** 2018-12-03

**Authors:** Fabrizio Sors, Franziska Lath, Alexandra Bader, Ilaria Santoro, Alessandra Galmonte, Tiziano Agostini, Mauro Murgia

**Affiliations:** 1 Department of Life Sciences, University of Trieste, Trieste, Italy; 2 Department of Medicine, Surgery and Health Sciences, University of Trieste, Trieste, Italy; University of Rome, ITALY

## Abstract

In light of the growing body of research that is revealing the significant role of the auditory domain in sport, the present study aims to investigate the contribution of early auditory and visual information to the prediction of volleyball serves’ length. To this purpose, three within-subjects experiments were run, which differed among them in terms of stimuli (audiovisual congruent vs audiovisual incongruent; audio only vs video only) and/or in terms of number of possible answers. In particular, expert volleyball players were asked to predict the length of temporally occluded overhand serves, choosing among either two or three possible landing sectors. Response accuracy and response times were measured. For the incongruent stimuli, the results revealed that the percentage of predictions in line with early auditory information was significantly higher than the respective percentage of predictions in line with early visual information. For unimodal stimuli, prediction accuracy was significantly higher on the basis of auditory information than on the basis of visual information, without any difference on response times. Taken together, the results highlighted the relevance of early auditory information for the prediction of volleyball serves’ length.

## Introduction

In ball sports like volleyball and tennis, the serve has a key role. Indeed, if you perform it well, you can either directly score a point (i.e., an ace), or hinder the response of your opponent(s), thus having an advantage in the continuation of the rally. On the other hand, the receiver has to do her/his best to prevent these two possibilities from happening, trying at the same time to perform an effective side out. To this purpose, beside the importance of technical and tactical skills, it is fundamental to be able to accurately perceive the ball motion, in order to anticipate the appropriate position in time [1,2].

In this regard, previous research concerning various sports highlighted that athletes can infer the trajectory of the ball not only by its motion [3], but also from the visual information provided by the interaction between the opponent and the ball itself [4–8]. In particular it was observed that, in order to perform successful interceptions, the integration of early visual information from the kinematics of the opponent and from the ball flight is needed [9–11]. The effective interpretation of such an early information is facilitated by expertise, which promotes fast and accurate predictions concerning actions’ outcomes [12–16], also when anticipating on the basis of contextual information alone [17].

Thus, the importance of visual information in sport anticipation is well-documented and well-established. However, there is a growing body of research revealing that also auditory information has a relevant role in sport [18]. In this regard, the first known evidence was provided by Takeuchi [19], who observed that auditory deprivation significantly hinders tennis performances, especially in terms of receiving and returning the serve. Another demonstration was provided by Brown, Kenwell, Maraj, and Collins [20], who observed that an increase of the gunshot’s intensity promotes a decrease of sprinters’ reaction times. Subsequently, some studies revealed that, on the one hand, sports-related sounds activate premotor and motor brain areas on the basis of expertise [21], and that, on the other hand, athletes can discriminate the sound they produce while performing specific sport movements from the sound produced by other athletes performing the same movements [22,23]. Another series of studies highlighted that an appropriate use of auditory information–either as a model [24], or as a feedback [25]–significantly improves performance (for a review, see [26]).

Some recent research revealed that, by means of auditory information alone, athletes are able to detect the movement intentions of an opponent in basketball [27] as well as to discriminate among different types of fencing attacks [28]. Moreover, it has been observed that the manipulation of the sound intensity of tennis strokes significantly influences predictions of their outcome [29]. Of particular interest for the present study is the work by Sors and colleagues [30], who investigated the contribution of early auditory and visual information to the discrimination of shot power in soccer and volleyball. To this purpose, they created a task based on a two-alternative forced choice paradigm, through which participants had to discriminate the power of temporally occluded penalties/smashes presented in pairs, relying either on audiovisual information, or only on one of the two sources of information at a time. The results revealed that, for this specific task, early auditory information provided more relevant perceptual cues than the respective visual information, promoting faster responses, and in the case of volleyball also more accurate responses. Notwithstanding the original results provided by the authors, one weakness of their study was that the task they used was not very realistic, as acknowledged also by the authors themselves.

Considering the novelty of Sors and colleagues’ [30] results, as well as their potential impact on both research and applied contexts, we decided to further investigate on these issues by using a more realistic task, that more closely resembled a field performance situation. In particular, we asked participants to predict the landing zone of volleyball serves with respect to their length. This task was chosen as it requires an implicit estimation of shot power while engaged in a typical field performance situation, namely, predicting the length of a serve. We focused on the volleyball serve because it is a fundamental phase of the game. Indeed, the defending team has to prevent the ball from touching the floor and has to accurately receive the ball in order to prepare the ground for an effective attack. Thus, correctly predicting the landing zone of the ball is crucial for receivers.

Given the importance of predicting the landing zone of the ball, we conducted three experiments in order to investigate the role of early auditory and visual information in predicting the length of volleyball serves. What differentiated the experiments among them was the type of stimuli and the number of possible answers: in the first experiment, we used audiovisual stimuli, with auditory and visual information either congruent or incongruent with each other, and two possible answers; in the second experiment there were always two possible answers, but participants could rely only on one of the two sources of information at a time; finally, in the third experiment participants could rely only on one of the two sources of information at a time, and there were three possible answers.

## Experiment 1

The aim of Experiment 1 was to investigate the relevance of early auditory and visual information for the prediction of the length of volleyball serves in audiovisual stimuli. To this purpose, audio and video recordings of overhand serves were assembled either congruently or incongruently (i.e., the audio of a short serve combined with the video of a long serve, or vice versa), and then administered to participants, whose task was to predict the landing zone of the serves.

To the best of our knowledge, the only study which investigated on similar issues was that of Sors and colleagues [30], even though the task and the sport situations were different. In the audiovisual condition of that study participants performed above the chance level, so we hypothesized a similar performance level for the congruent trials of the present experiment. As regards the incongruent trials, responses cannot be classified as correct or wrong, but only as consistent either with the auditory or with the visual information. On the basis of the higher relevance of the auditory information observed by Sors and colleagues, we hypothesized that participants’ prediction would be more in line with auditory information than with visual information. Finally, we hypothesized slower response times in the incongruent trials than in the congruent ones, because of the conflict between auditory and visual information [31].

### Materials and methods

#### Participants

Twenty-one volleyball players (15 females, 6 males) participated in this experiment. They had an average age of 23.7 years (SD = 4.6) and an average playing experience in amateur leagues of 9 years (SD = 4.1); nineteen of them were right-handed, and two were left-handed. All participants had normal or corrected-to-normal vision, and reported no hearing disturbances. Moreover, a right-handed volleyball player aged 23 and with a playing experience of 12 years in amateur leagues was also recruited; he acted as a server in the stimuli recording phase. This individual has given written informed consent (as outlined in PLOS consent form) to publish both these details and the pictures portraying him in the “stimuli editing” section.

The protocol was approved by the Ethics Committee of the University of Trieste (Protocol 45, date 05.11.2012). The experiment was carried out in accordance with the recommendations of the Ethics Committee, with written informed consent obtained from each participant prior to the beginning of the experiment.

#### Apparatus

An action camera with a temporal resolution of 60fps and a spatial resolution of 1080p (GoPro HD Hero 3 Black Edition) was used to record the visual stimuli. A stereo microphone (Soundman Binaural, OKM II Professional) connected to an external sound card (M-AUDIO MobilePre) was used to record the auditory stimuli. The programs iMovie and Adobe Audition 3.0 were used to manipulate the video and audio recordings, respectively; iMovie was also used to assemble audio and video recordings.

The E-prime Professional 2.0 software was used to program the experiment. A laptop computer Dell Inspiron 5559 with a 15.6” LED display was used to run the experiment; Sennheiser HD 205 II circumaural headphones were used to convey auditory stimuli.

#### Stimuli recording

**Fig 1:**
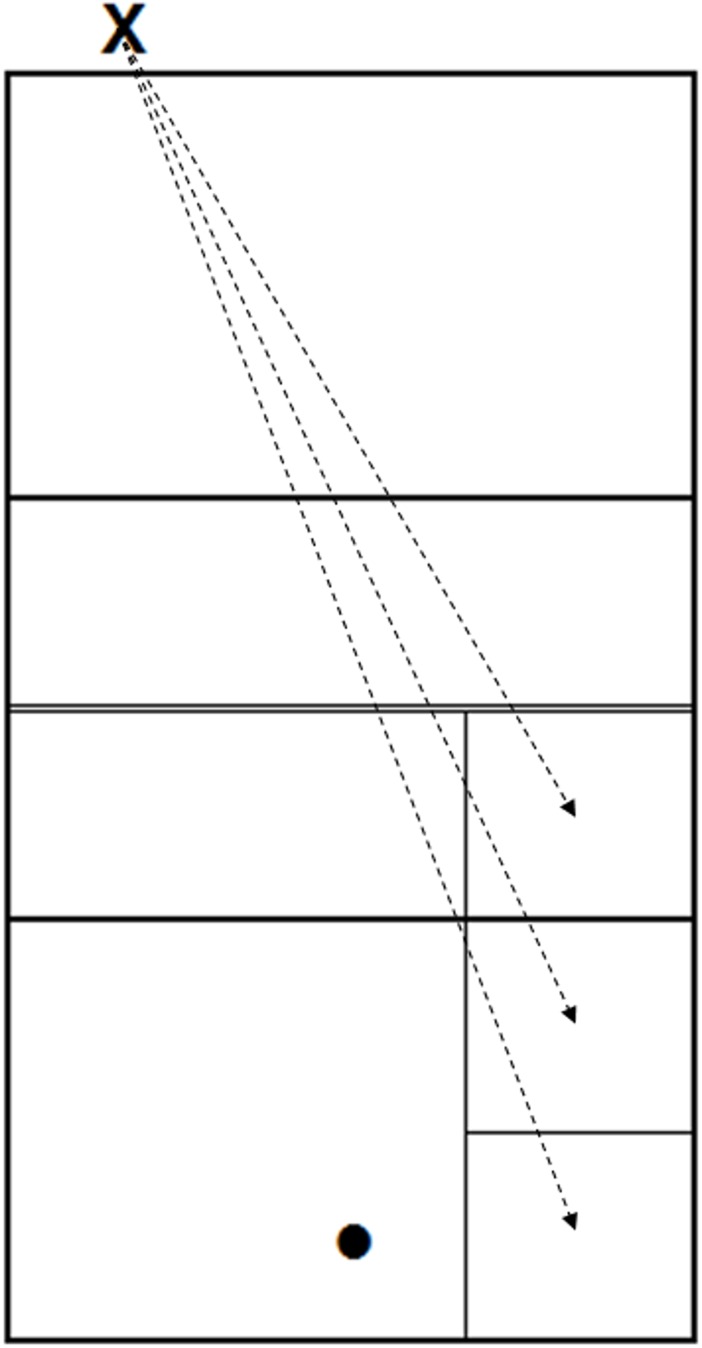
Reproduction of the recording setting. The “X” represents the serving point; the arrows indicate the direction of the serves, and the squares represent the three landing sectors. The dot indicates where the camera and the microphone were placed.

The stimuli were recorded on a regular volleyball court. Observing Fig 1, the serves were performed right behind the end-line, 1.5 m away from the side-line, and were directed toward the opposite side-line of the other half of the court; in this area, three sectors of 3 x 3 m were delimited with some adhesive tape, in order to identify the ball landing zone. A tripod with the action camera and the stereo microphone was placed inside the court, 1.5 m from the end-line, centred with respect to the width of the court. The camera and the microphone were at a height of 1.75 m and were oriented straight on, reproducing the possible perspective of a player waiting to receive a serve. To record the stimuli, the server was asked to perform several overhand serves aiming at the three delimited sectors. Overall, 90 serves were recorded.

#### Stimuli editing

The first operation made on the serves database was to discard those which did not land in one of the three delimited sectors, as well as those which had a disturbance either in the audio file (e.g., voice/noise echoes) or in the video one (e.g., the net moving because a previous serve touched it). After this operation, the database consisted of 48 serves. Out of these 48 serves, 20 were selected, half of which landing in the sector near the net (short serves), and the other half landing in the sector near the bottom of the court (long serves). An expert coach assisted the selection, suggesting the exclusion of some serves on the basis of their technical execution.

The audio and video files of these serves were edited and assembled through the above mentioned software. Two kinds of stimuli were created: 1) congruent stimuli, in which the video and the audio files of the same serve were assembled; 2) incongruent stimuli, in which the video of a short serve was assembled with the audio of a long serve, and vice versa. Altogether, 40 stimuli were created: 20 were congruent (10 short and 10 long serves) and 20 were incongruent (10 with the video of a short serve combined with the audio of a long serve and 10 with the video of a long serve combined with the audio of a short serve). Moreover, 8 other stimuli (4 congruent and 4 incongruent) were created for the practice trials, assembling the audio and video files of 4 of the serves that were not previously selected.

**Fig 2:**
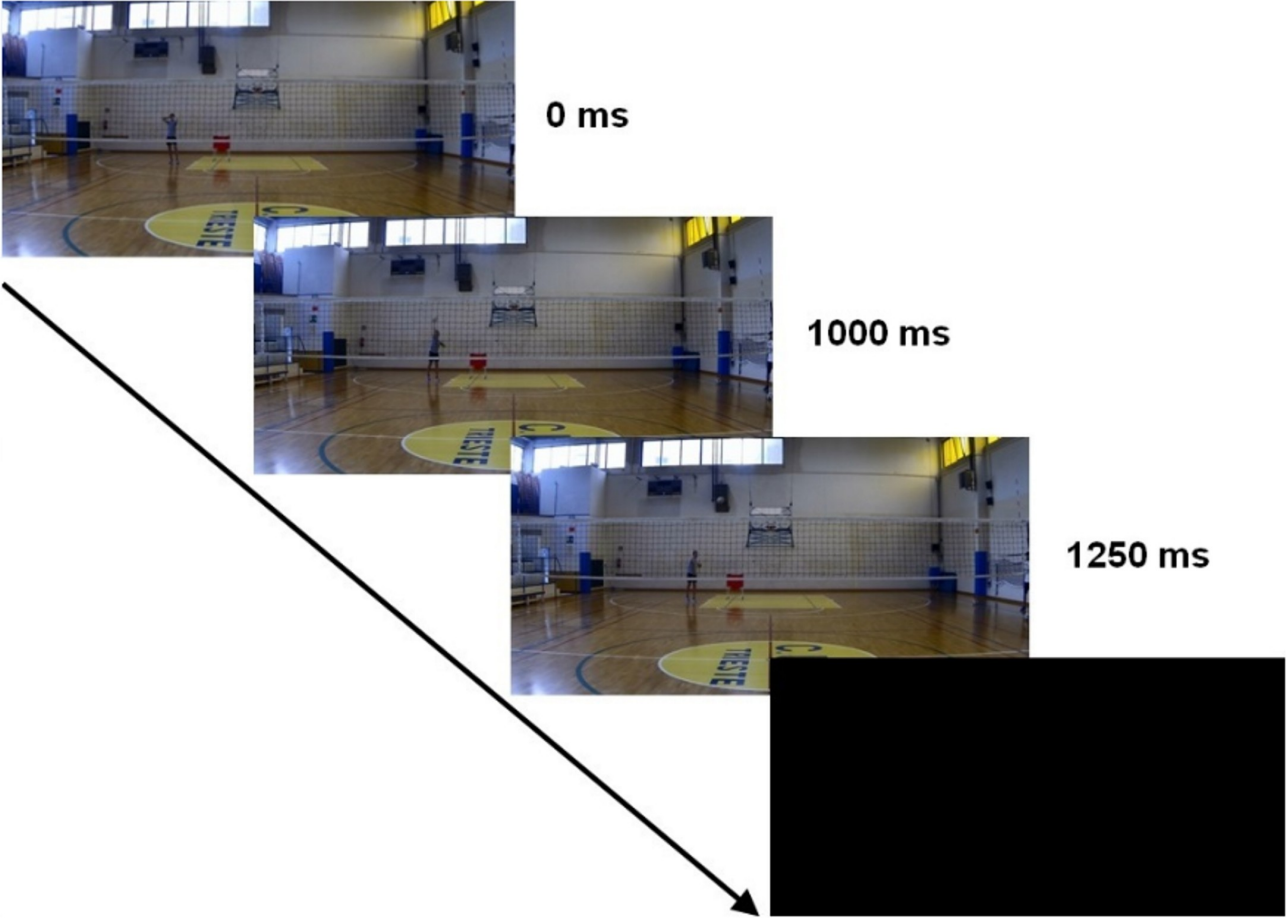
The visual part of a prototypical stimulus. The audio was synchronized with the video, and they were either congruent or incongruent between them as concerns the length of the serve.

The duration of every stimulus was 1250 ms, using as a reference point the impact between the hand of the server and the ball, which occurred always 1000 ms after the beginning of the stimulus. As a consequence, every stimulus included the whole serve movement (including the toss) and the first 250 ms of the ball flight; at this point, the video was occluded with a black screen, and the audio was interrupted (see Fig 2)

#### Procedure

Participants were individually tested in a quiet room. The distance from the screen was 40 cm, thus the visual angle covered by the server (from the feet to the head) was around 5.3°. The volume of the auditory stimuli was set at a comfortable level by the experimenters and was kept constant for all participants.

**Fig 3:**
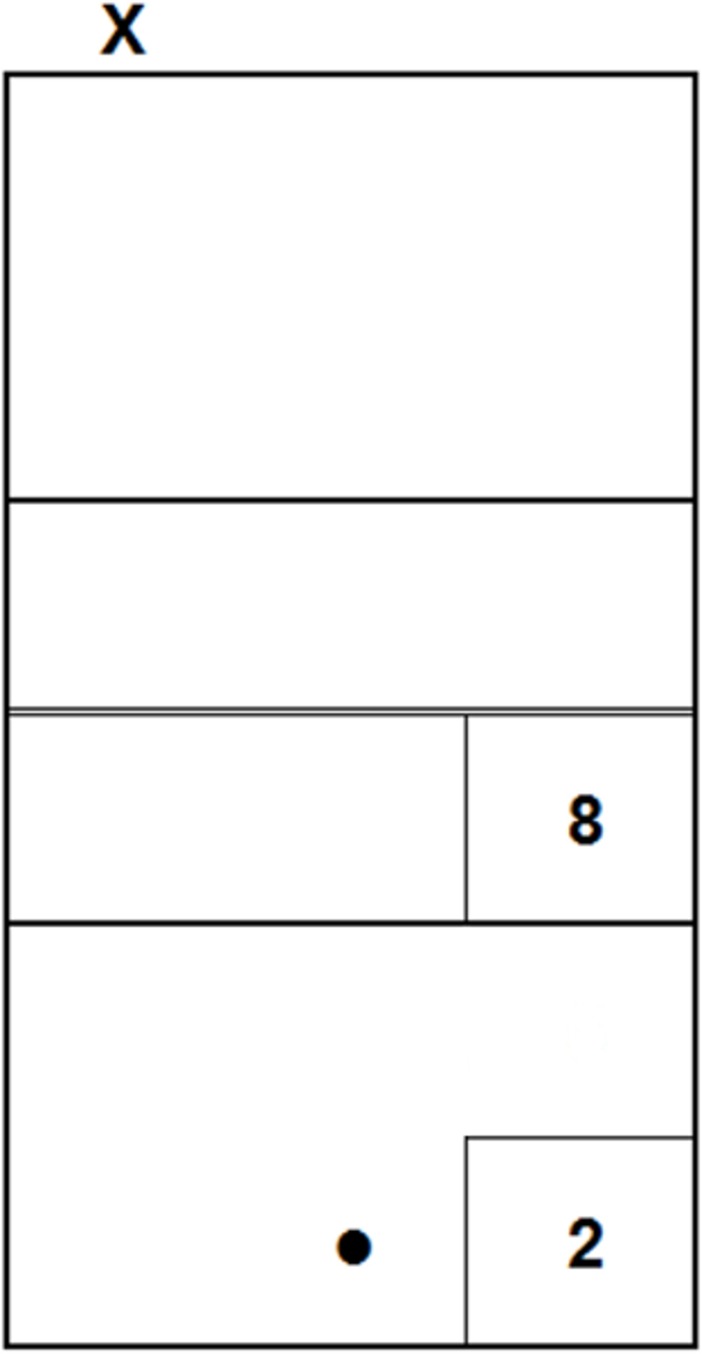
Correspondence between the sectors and the response keys on the keypad. This image was included in the instructions.

Upon their arrival, participants were asked to seat in front of the laptop and to wear the headphones; then, the experimenter launched the experimental session. The session started with 8 practice trials, and then consisted of two blocks, each composed of 20 test trials, for a total of 40 test trials. Out of these 40 trials, 20 were congruent (10 short and 10 long serves) and 20 were incongruent (10 with the video of a short serve combined with the audio of a long serve and 10 with the video of a long serve combined with the audio of a short serve). In order to standardize the instructions, the following text–together with the image reproduced in Fig 3 –was presented at the beginning of the session:

You will see some volleyball serves, performed from the point marked with the "X", and recorded from the point marked with the dot. The movies stop soon after the ball is hit by the player.

Your task is to predict in which sector the ball will land, by pressing the respective number on the keypad, always starting from key "5". Wait the end of each movie to make your prediction, trying to be as accurate and fast as possible.

Thus, the participants’ task was to predict in which of the two sectors each serve would land by pressing one of two keys–namely, “8” for short serves or “2” for long serves (see Fig 3)–on the keypad of a QWERTY keyboard. These keys were chosen because of the consistency of their position on the keypad with that of the sectors on the court from the participants’ perspective. After each response, there was an interval of 1500 ms before the beginning of the subsequent stimulus.

For the congruent trials, we evaluated participants’ performance in terms of response accuracy and response times; for the incongruent trials, we measured the percentage of responses consistent with auditory information (and consequently, by subtraction, the percentage of those consistent with visual information), as well as response times. Responses faster than 150 ms (accounting for less than 1% of the data collected) were excluded from the analyses.

#### Statistical analyses

We conducted a paired samples t-tests to compare response times between congruent and incongruent trials. Then, for congruent trials, we calculated the *d’* scores for each participant as a measure of response accuracy and conducted a one-sample t-test against the chance level. Analogously, we calculated the *d’* scores also for the incongruent trials to test for the presence of a response bias in favour of auditory information.

### Results and discussion

**Fig 4:**
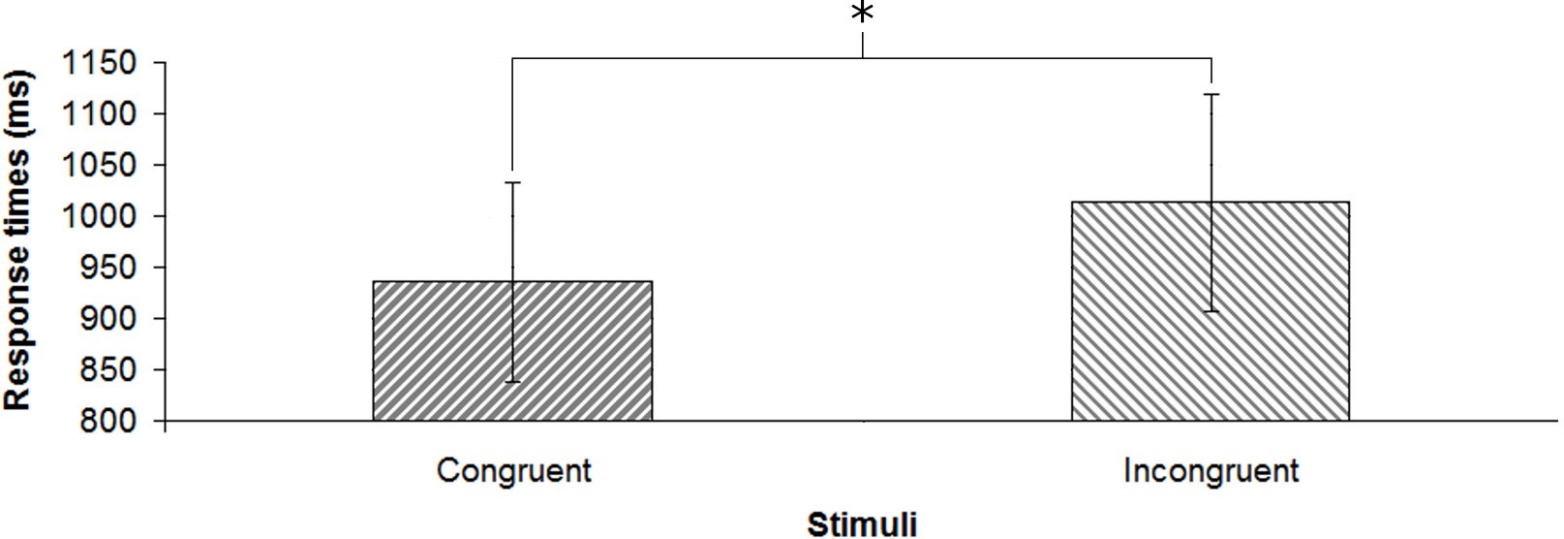
Response times for the two kinds of stimuli. Error bars show the standard error of the mean; * denotes statistical significance.

**Fig 5:**
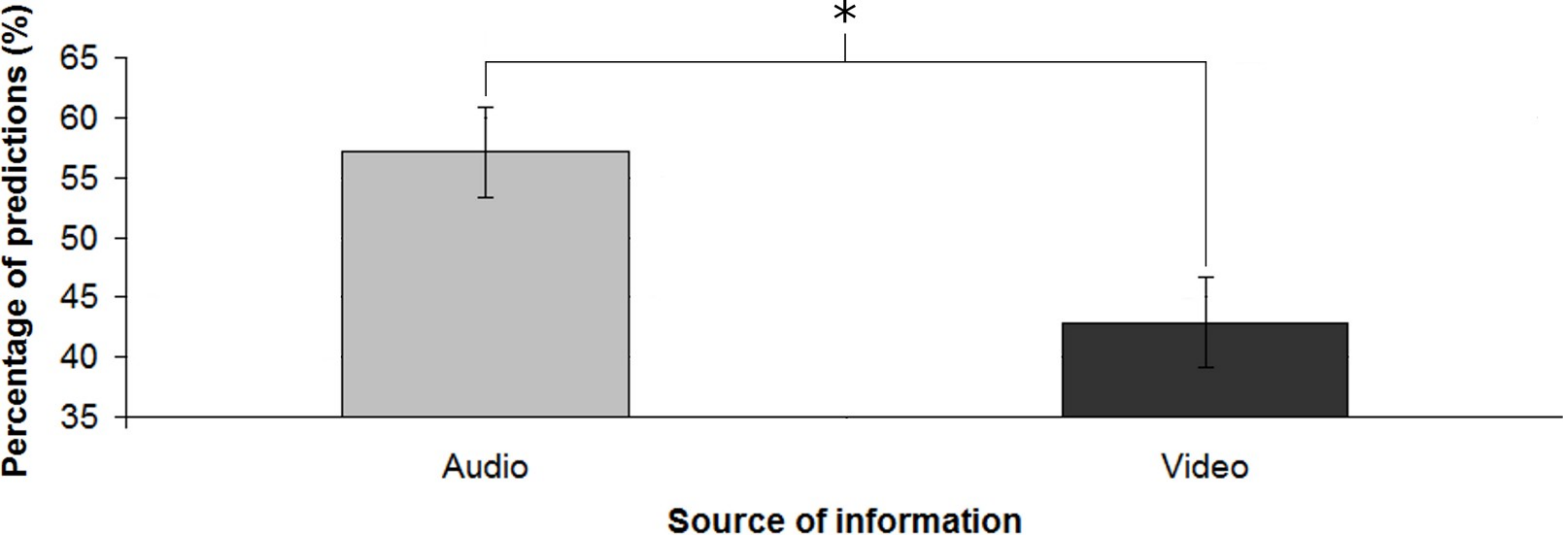
Percentage of predictions in line with the two sources of information for the incongruent stimuli. Error bars show the standard error of the mean; * denotes statistical significance.

Concerning response times (Fig 4), participants were significantly slower in the incongruent trials (M = 1013.29 ms, SD = 488.01 ms) than in the congruent ones (M = 935.43 ms, SD = 449.02 ms) [*t*(20) = 2.296; *p* < 0.05; *d* = 0.17]. For congruent trials, participants did not perform above the chance level (M = 47.86%, SD = 15.78%). For incongruent trials (Fig 5), the predictions in line with auditory information (M = 57.14%, SD = 17.22%) were significantly higher than the predictions in line with visual information (M = 42.86%, SD = 17.22%) [*d’* = .52; *t*(20) = 2.096; *p* < 0.05; *d* = 0.46].

Consistently with our hypothesis on responses times, the results revealed that participants were slower in making their predictions for the incongruent stimuli compared to the congruent ones; this was reasonably due to the conflict between auditory and visual information. For the congruent stimuli, participants’ prediction accuracy was not different from the chance; we can hypothesize that this outcome was due to the difficulty of the task, whose accurate execution might require more information than the early one provided to participants in the present experiment. Finally, for the incongruent stimuli the results revealed that participants’ predictions were more in line with early auditory information than with early visual information, consistently with the results of Sors and colleagues [30].

The results for incongruent trials can be interpreted in different ways: on the one hand, it is possible that athletes relied more on auditory information than on visual information; on the other hand, it is possible that they relied on visual information, but misinterpreted it. The results for congruent trials suggest that early information might be insufficient to promote an accuracy higher than the chance. However, we cannot exclude that some early information can be used to discriminate between long and short serves and that the poor performance we observed in Experiment 1 derives from the interference of one source of information over the other one. As a consequence, in Experiment 2 we investigated auditory and visual information separately.

## Experiment 2

Experiment 2 was conceived to better understand the contribution of early auditory and visual information to the prediction of volleyball serves’ length. To this purpose, participants were required to perform the same task of Experiment 1, but in this case they could rely either on auditory information alone or on visual information alone. In light of the results of Sors and colleagues [30], we hypothesized to observe both faster and more accurate predictions when participants could rely on auditory information than when they could rely on visual information.

### Materials and methods

#### Participants

Twenty-one volleyball players (16 females, 5 males), different from those who participated in Experiment 1, took part in the present experiment. They had an average age of 24.2 years (SD = 5.8) and an average playing experience in amateur leagues of 9.5 years (SD = 4.2); eighteen of them were right-handed, and three were left-handed. All participants had normal or corrected-to-normal vision, and reported no hearing disturbances.

The protocol was approved by the Ethics Committee of the University of Trieste, and the experiment was carried out in accordance with its recommendations. Written informed consent was obtained from each participant prior to the beginning of the experiment.

#### Stimuli

The same 20 serves selected for Experiment 1 were also considered for the present experiment. However, in this case the audio and video files were edited separately, thus creating two kinds of stimuli–auditory and visual–for each serve. Moreover, the files of 4 serves among the non-selected ones were used to create the stimuli for the practice trials. Like in the previous experiment, every stimulus lasted 1250 ms, including the whole serve movement and the first portion of the ball flight.

#### Procedure

The participants’ task was the same as for Experiment 1, namely, predicting the landing sector of each serve. Every experimental session started with 4 practice trials, and then consisted of two blocks, each composed of 20 test trials. The experimental conditions were two: Audio (auditory information alone), and Video (visual information alone). Thus, as a whole, each participant was administered 80 test trials: 40 in the Audio condition and 40 in the Video condition. A within subjects experimental design was used, with the participants assigned to the two conditions in a counterbalanced order. The dependent variables were response accuracy and response times; as for Experiment 1, responses faster than 150 ms were excluded from the analyses.

The procedure was similar to that of the previous experiment. Upon their arrival, participants were asked to seat in front of the laptop and to wear the headphones (also in the Video condition, so that they were in the same situation in both conditions). Then, the experimenter launched the first experimental session; the second session was administered 5 minutes after the conclusion of the first one, to provide participants with an appropriate rest between the two sessions.

#### Statistical analyses

To compare participants’ response accuracy in the two conditions against the chance level, we conducted two one-sample t-tests. Then, we conducted a paired sample t-test to compare response accuracy between the two conditions, and another one to compare response times between the two conditions.

### Results and discussion

**Fig 6:**
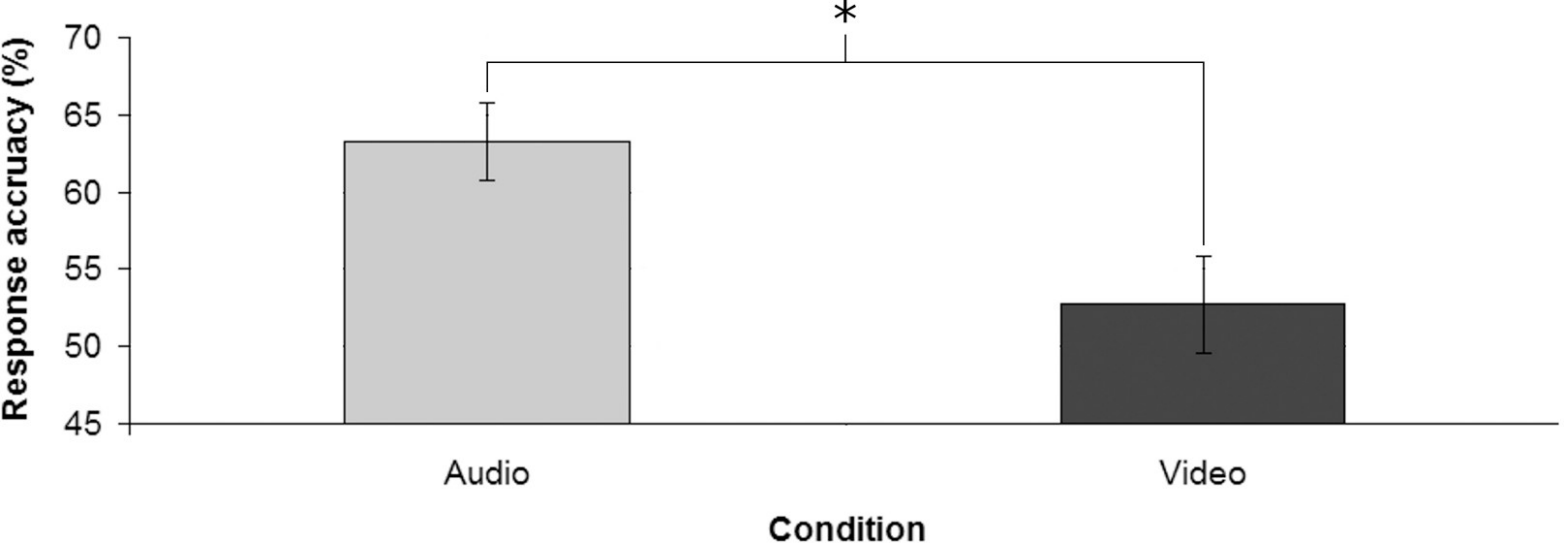
Response accuracy in the two conditions. Error bars show the standard error of the mean; * denotes statistical significance.

Participants response accuracy (Fig 6) was significantly above the chance level in the Audio condition (M = 63.24%, SD = 11.48%) [*t*(20) = 5.286; *p* < 0.001; *d* = 1.15] but not in the Video condition (M = 52.71%, SD = 14.36%). Moreover, response accuracy was significantly higher in the Audio condition compared to the Video condition [*t*(20) = 2.289; *p* < 0.05; *d* = 0.81]. No significant difference in the response times between the two experimental conditions emerged (MAudio = 778.86 ms, SDAudio = 299.08 ms; MVideo = 674.86 ms, SDVideo = 235.35 ms).

The results highlighted that, as hypothesized, participants were more accurate when they could rely on auditory information than when they could rely on visual information; moreover, in the former case the performances were also above the chance level, while in the latter case they were not. For response times, differently from our hypothesis, the results revealed no difference between the two conditions.

Taken together, the results of Experiment 2 suggest that, at a comparable elaboration time, early auditory information would provide more relevant cues than the respective visual information to predict the landing zone of overhand serves with respect to their length. Indeed, auditory information allowed a prediction accuracy above chance, while visual information did not.

## Experiment 3

Experiment 3 is almost a replication of Experiment 2; the novelty of the present experiment is that, in order to have a more difficult task resembling even more closely a realistic situation, we considered also serves landing in the middle sector. In light of the results of Experiment 2, we hypothesized to observe more accurate predictions when participants could rely on auditory information than when they could rely on visual information, without any difference concerning response times. Moreover, we also hypothesized that accuracy would be above the chance in the Audio condition, but not in the Video condition. Should the results be replicated with such a more difficult task, they would bring further support to the relevance of early auditory information in predicting serves’ length, as compared to the respective visual information.

### Materials and methods

#### Participants

Seventeen volleyball players (7 females, 10 males), different from those who participated to the previous experiments, took part in the present experiment. They had an average age of 26.3 years (SD = 6.8) and an average playing experience in amateur leagues of 8.7 years (SD = 4.8); sixteen of them were right-handed, and one was left-handed. All participants had normal or corrected-to-normal vision, and reported no hearing disturbances.

The protocol was approved by the Ethics Committee of the University of Trieste, and the experiment was carried out in accordance with its recommendations. Written informed consent was obtained from each participant prior to the beginning of the experiment.

#### Stimuli

Compared to Experiments 1 and 2 –in which only two sectors were considered–in the present experiment we added a third sector, namely the middle one. Thus, besides the 10 short serves and the 10 long ones considered for the previous experiments, 10 serves landing in the middle sector were also selected. The audio and video files of these 10 more serves were edited as for Experiment 2, that is, creating two kinds of stimuli–auditory and visual–for each serve. Moreover, the files of 2 more serves among the non-selected ones were used to create the stimuli for the practice trials. Like in the previous experiments, every stimulus lasted 1250 ms, including the whole serve movement and the first portion of the ball flight.

#### Procedure

**Fig 7:**
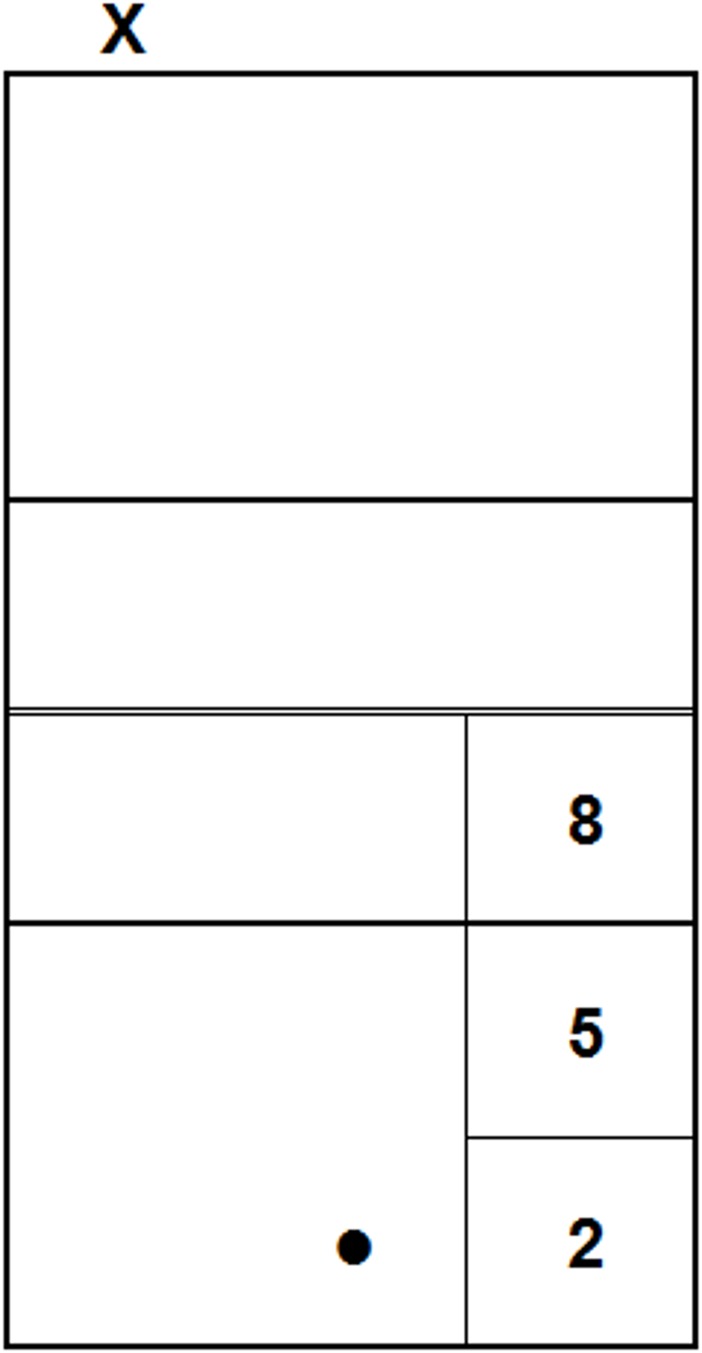
Correspondence between the sectors and the response keys on the keypad. This image was included in the instructions.

The participants’ task was similar to that of the previous experiments, that is, predicting the landing sector of each serve. However, in Experiment 3 participants had one more response option: beyond keys “8” and “5”, there was also key “5” for intermediate serves (see Fig 7 for the correspondence between the sectors and the keys).

Every experimental session started with 6 practice trials, and then consisted of two blocks, each composed of 30 test trials. The experimental conditions were two: Audio and Video. Thus, as a whole, each participant was administered 120 test trials: 60 in the Audio condition and 60 in the Video condition. The experimental design, the dependent variables, and the procedure were the same as for Experiment 2.

#### Statistical analyses

The analyses conducted were the same as for Experiment 2.

### Results and discussion

**Fig 8:**
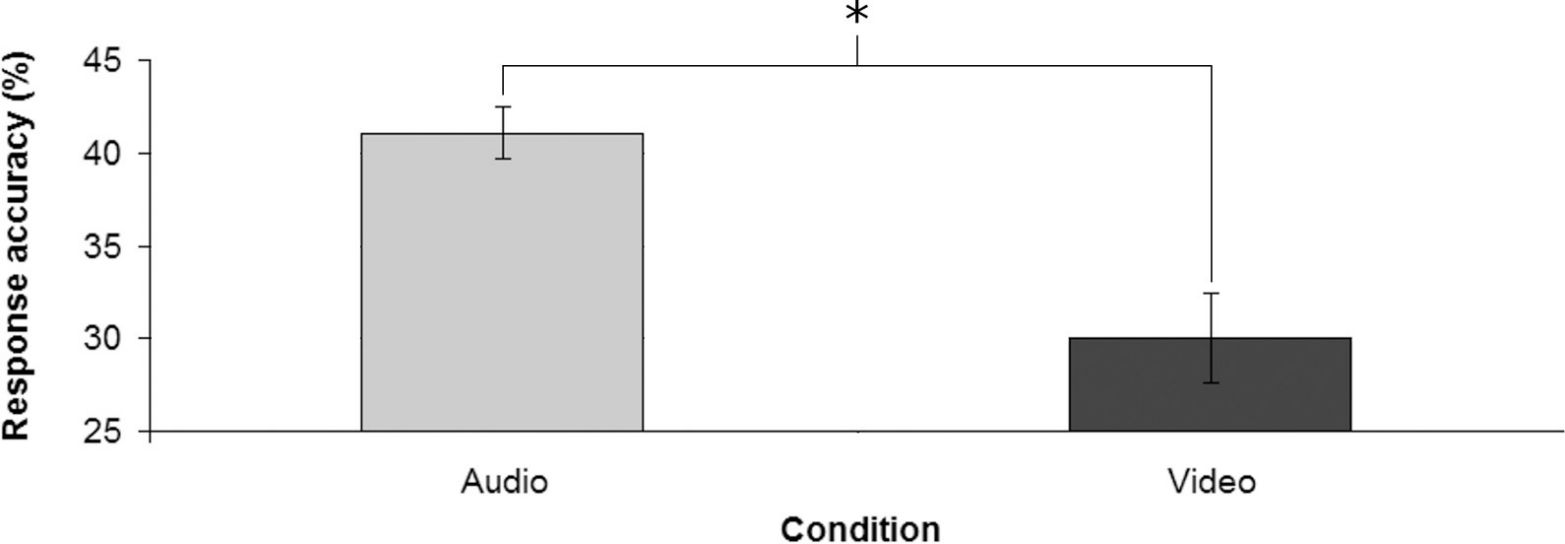
Response accuracy in the two conditions. Error bars show the standard error of the mean; * denotes statistical significance.

Participants response accuracy (Fig 8) was significantly above the chance level in the Audio condition (M = 41.08%, SD = 5.80%) [*t*(16) = 5.504; *p* < 0.001; *d* = 1.33] but not in the Video condition (M = 30.00%, SD = 9.96%). Moreover, response accuracy was significantly higher in the Audio condition compared to the Video condition [*t*(16) = 3.951; *p* < 0.01; *d* = 1.36]. No significant difference in the response times between the two experimental conditions emerged (MAaudio = 1206.32 ms, SDAudio = 694.44 ms; MVideo = 1243.31 ms, SDVideo = 379.10 ms).

As hypothesized, participants performed better in the Audio condition than in the Video condition, without any difference concerning response times; moreover, also in the present experiment the performance in the former condition was above the chance level, while in the latter condition it was not.

Taken together, the results of Experiment 3 bring further support to the claim that early auditory information would be more relevant than the respective visual information for the prediction of volleyball serves’ length. Indeed, also in a more difficult task, auditory information promoted a better-than-chance performance, while visual information did not.

## General discussion

Previous research on anticipation in sport focused almost exclusively on the visual domain, highlighting the importance of visual information, especially in ball sports [4,6]. However, in recent years the relevant role of auditory information has started to emerge, both as a means for performance improvement [26] and as a precious source of cues for anticipation [27–29]. In this regard, Sors and colleagues [30] observed that, to discriminate the power of soccer penalty kicks and volleyball smashes, early auditory cues are more informative than the respective visual cues. The aim of the present study was to investigate whether similar results could emerge when using a task that more closely resembles a field performance situation, that is, predicting the landing zone of volleyball serves with respect to their length.

To reach this aim, in Experiment 1 participants were provided with stimuli that were characterized either by the congruence between auditory and visual information, or by the incongruence between them. The results highlighted that the majority of predictions for incongruent stimuli was in line with auditory information, rather than with visual information. The accuracy in response to congruent stimuli was not different from chance, thus suggesting that early information might not be sufficient to perform this task effectively.

To better understand the contribution of early auditory and visual information, in experiments 2 and 3 participants were forced to rely either on the former or on the latter at a time to make their predictions. The results revealed not only that auditory information promoted a higher prediction accuracy compared to visual information, but also that by means of auditory information the accuracy was above chance while by means of visual information it was not. As a consequence, it is possible to claim that auditory information alone can be somehow used to predict the length of overhand serves at early stages of information acquisition.

There is an apparent inconsistency between the results of Experiment 1 and those of experiments 2 and 3. In the congruent condition of the first experiment, participants could rely on both sources of information, yet the prediction accuracy was not different from chance. Instead, in the latter experiments participants could rely on only one source of information at a time: in spite of this reduced amount of information, by means of auditory information the prediction accuracy was above the chance, while by means of visual information it was not. Thus, the apparent inconsistency between the results of Experiment 1 and those of experiments 2 and 3 could be explained in terms of a potentially misleading effect of early visual information, which might have eliminated the advantage of the respective auditory information in the congruent condition of Experiment 1.

From a broader perspective, for the specific task required, it seems that early visual information (i.e., the kinematics of the server and the first portion of the ball flight) is still ambiguous with respect to the outcome of the action, while this is not to the case for early auditory information (i.e., the impact with the ball and its echo). As a consequence, the present study shows a superior contribution of auditory information compared to that of visual information at early stages of information acquisition. It is evident that visual information becomes more accurate over time, however until a certain moment (i. e., 250 ms after the hand-ball impact) auditory cues seem more informative than visual cues. Interestingly, the present study extends the results of Sors and colleagues [30] to a more realistic task, suggesting that the auditory advantage is not limited to the discrimination of shot power but is also present in a task requiring an implicit estimation of shot power, that is, the prediction of serves length.

The present study further contributes to the development of a very recent line of research highlighting the role of auditory information in action anticipation. Indeed, in the last two years different studies demonstrated that opponents’ behaviours can be predicted also on the basis of auditory information. This has been observed in various sports: for instance, it has been shown that basketball players are able to detect the movement intentions of an opponent [27]; fencers can discriminate among different types of fencing attacks [28]; and tennis players differently predict the length of strokes depending on the sound intensity [29]. Within this line of research, the current study reveals that early auditory information can provide useful cues for the anticipation of volleyball serves.

From an applied perspective, during competitions there are some situations in which visual information may not be completely available–or not available at all–to athletes. For example, in the volleyball serve the opponents can more or less intentionally hide the server to the eyes of the receivers, thus hindering the perception of relevant visual information; another example is given by soccer free kicks, in which goalkeepers may have difficulties in seeing the run-up of the kicker and the beginning of the ball flight due to the wall. The results of the present study–as well as those of previous studies of this line of research–suggest that in similar situations auditory information could provide useful cues, thus athletes may benefit from being trained to appropriately interpret such auditory cues in real settings. Therefore, beyond continuing to investigate the relevance of auditory and visual information in sport, it would be both interesting and potentially useful to develop and test the effectiveness of new kinds of perceptual-motor training based on the outcomes of basic research. Similar training protocols already exist and have proven to be effective in ball sports, but most of them are focused on the visual domain [32–35], while the audio-based interventions described in literature have rarely focused on ball sports (for a review, see [26]).

Like every study, the present work does have some limitations. Typically, in these studies experimenters try to reproduce the conditions that athletes have to cope with in real life, but every manipulation necessarily affects the ecologicity of the situation. In our case, we used computer-based tasks reproducing a simplified setting, in which some factors that could potentially influence visual information (e.g., other players) and auditory information (e.g., crowd noise) were absent. We made sure that the visual angle covered by the server on the monitor was comparable to that of a server from a distance of about 18 meters; however, we acknowledge that the use of wider screens, 3D displays and/or virtual reality would contribute to better understand the role of visual information [36]. Indeed, previous studies highlighted that the size of the screen can affect gaze behaviour of participants [37], and that the two-dimensional display, as opposed to the 3D aspect of live-action settings, can induce different visual search behaviours [38]. As auditory information is less studied, the potential existence of similar discrepancies between real and digitally reproduced sport stimuli for the auditory modality has not been investigated yet; however, we are aware that auditory stimuli more closely resembling the real sounds can also be created. In particular, this could be done by using advanced recording and reproduction instruments/techniques, for instance dummy head microphones and 3D reproduction systems. To date, the majority of studies investigating similar issues used instruments comparable to ours; future studies should take advantage of the continuous advances in technology to create experimental settings as realistic and immersive as possible.

## Conclusions

To sum up, the results of the present study highlighted that, to predict the length of overhand serves, early auditory information would provide more relevant perceptual cues than early visual information, promoting a higher prediction accuracy. These outcomes bring further support to the literature that, in recent years, has revealed the relevant role of auditory information in the perception and execution of complex movements [39–44]. Previous studies demonstrated that some perceptual-motor phenomena traditionally studied from the visual perspective have an auditory counterpart (for a review, see [45]). In the present study we showed that the prediction of the landing zone of the ball–which has commonly been investigated focusing on visual cues–can be investigated also focusing on auditory cues. The outcomes of this first attempt revealed that, as concerns the length of the serves, the latter are even more informative than the former. Given the lack of studies on the contribution of auditory information to sport anticipation, further research is necessary to provide more solid evidence on it. More in general, future studies on similar issues should consider the potential relevance of auditory information and its interaction with visual information in ecological sport situations.

## Supporting information

S1 File Datasets. (XLS)
